# Effects of different exercise programs and minimal detectable changes in hemoglobin A1c in patients with type 2 diabetes

**DOI:** 10.1186/s13098-016-0123-y

**Published:** 2016-02-16

**Authors:** Carlos Gabriel de Lade, João Carlos Bouzas Marins, Luciana Moreira Lima, Cristiane Junqueira de Carvalho, Robson Bonoto Teixeira, Maicon Rodrigues Albuquerque, Janice Sepúlveda Reis, Paulo Roberto dos Santos Amorim

**Affiliations:** Physical Education Department, Universidade Federal de Viçosa, Av. PH Rolfs, s/n. Campus Universitário, Viçosa, MG 36570-900 Brazil; Medicine and Nursing Department, Universidade Federal de Viçosa, Viçosa, MG Brazil; Santa Casa de Belo Horizonte, Rua Domingos Vieira, 300/504, Santa Efigênia, Belo Horizonte, MG 30150-240 Brazil

**Keywords:** Diabetes *mellitus*, Hemoglobin A1c, Strength training, Aerobic training, Minimum detectable change

## Abstract

**Background:**

The incidence of diabetes mellitus is increasing worldwide, resulting in a global epidemic. The most common type, the type 2 diabetes *mellitus*, constitutes of 90–95 % of the cases and is characterized by the action of and/or impaired insulin secretion. Regular exercise is a recommended strategy in several studies and guidelines for type 2 diabetes control and complications associated with it. Therefore, we evaluated and compared the effects of aerobic and strength exercise programs on the glycemic control in patients with type 2 diabetes.

**Methods:**

The selected patients were divided into groups which performed moderate strength training (ST) and aerobic training (AT). The study lasted 20 weeks and was divided into two 10 week phases with anthropometric (body mass index, waist, abdomen and hips circumferences, waist/hip ratio) and biochemical (glycemic and lipid profile) assessments at baseline, 10 weeks and 20 weeks. For intra and inter analyses a mixed ANOVA model was used. Individual changes were calculated using the minimum detectable change, based on a 90 % confidence interval.

**Results:**

Eleven patients (five men and six women) completed the 20 weeks of training; five from the ST group and six from the AT. No significant changes were observed in any anthropometric variable in either group. Statistically significant differences were found in mean hemoglobin A1c in both groups between *baseline* (AT: 8.6 ± 2.5; ST: 9.2 ± 1.9) and 10 weeks (AT: 7.2 ± 1.7; ST: 7.9 ± 1.2) (p = 0.03), and *baseline* (AT: 8.6 ± 2.5; ST: 9.2 ± 1.9) and 20 weeks (AT: 7.5 ± 1.7; ST: 7.4 ± 0.9) (p = 0.01). For the minimal detectable changes, 40 % of the ST and 33 % of AT achieved these changes for hemoglobin A1c.

**Conclusion:**

Both aerobic and strength exercises can help the metabolic control in patients with type 2 diabetes, even without significant changes in anthropometry over the 20 weeks of training. However, this period was sufficient to cause changes in hemoglobin A1c values and the estimated average glucose, which are important parameters in controlling diabetes, thus signaling an important consequence of adhering to an exercise routine for type 2 diabetic patients.

**Electronic supplementary material:**

The online version of this article (doi:10.1186/s13098-016-0123-y) contains supplementary material, which is available to authorized users.

## Background

The incidence of diabetes *mellitus* (DM) is increasing worldwide, resulting in a global epidemic [1]. More than 382 million people are currently affected worldwide, with an increase to 592 million forecasted for the year 2035 [2, 3]. Yet, according to the International Diabetes Federation, Brazil is the fourth country in the world in number of people with DM, with almost 12 million patients [4].

The most common type, the type 2 diabetes *mellitus* (DM 2), makes up 90–95 % of the cases and is characterized by impaired action and/or insulin secretion. This disease is more common in people older than 45 years of age and overweight [2]. However, as a result of the increase of obesity among young people, DM 2 is becoming more frequent among children and young adults [5]. The increasing prevalence of DM 2 can also be attributed to other factors, such as population growth and ageing, greater urbanization, the increasing prevalence of obesity and physical inactivity, as well as increased survival amongst patients with DM [4, 6].

Regular exercise is a strategy recommended by a diverse number of studies, and a guideline to control DM 2 and its associated complications [3, 7–9] and, in association with food planning and drug therapy, it has been regarded as one of the three main components in the control and treatment of DM [10]. It is noteworthy that diabetes patients demonstrate less aerobic conditioning, and lower levels of muscular strength and flexibility when compared with unaffected individuals of the same age and gender. Diabetics that are physically active and/or present good aerobic conditioning feature better prognosis than those who are inactive and/or those with low aerobic conditioning [6].

Both aerobic and strength exercises can increase insulin action, improve the lipid profile, glycemic levels, blood pressure (BP), decrease the risk of cardiovascular diseases, lower body weight [11–13], reduction of mortality [14], prevent complications related to DM and improve the life quality of diabetics when performed continuously [12]. These factors make the inclusion of physical activity a fundamental element of a diabetic’s treatment.

As such, the present study aimed to evaluate and compare the effects of supervised aerobic exercise and strength programs on glycemic control in patients with DM 2 treated at the Hiperdia Center of Viçosa, MG.

## Methods

### Study design

The study was within and between subjects design which lasted 20 weeks and was developed as part of the project “Evaluation and Treatment of Diabetic and Hypertensive Patients Served by the Center Hiperdia of Viçosa”, a partnership of the Federal University of Viçosa with the program Hiperdia Minas. The study was conducted entirely at the Hiperdia Center in Viçosa/MG. The project was approved by the Committee of ethics in research with human beings at the Federal University of Viçosa, under the CAAE n^o^ 28144814.0.0000.5153, respecting the rules of Brazilian Legislation (resolution 12/466) on studies with humans.

After the treadmill test (TT) and before the start of the intervention program with exercise, all volunteers were undergoing anthropometric assessments and blood tests for metabolic control analysis.

The participants were randomly divided into two groups, with distinct exercise programs. One group performed aerobic training (AT) and the other strength training (ST). The study was divided into two phases, both with 10 weeks.

At the end of the first phase, the participants were subjected to the same evaluations as at the beginning of the program. After the first reevaluations (10 weeks), the participants started the second phase, with changes in programs and the intensity of the exercises. At the end of the second phase (20 weeks), the participants were subjected to the same initial assessments again. At the beginning of the study, all participants were told not to change their eating habits, unless prescribed a calorie reduced food plan. In addition, physicians were asked not to change any medication being given to the patients during the 20 weeks of training.

### Sample

Sampling of the present study was selected from the Viçosa Hiperdia Reference Center, which covers 9 cities in the Zona da Mata region of Minas Gerais. Among the 547 diabetic patients, assisted until April 2014, 95 patients were pre-selected through a registration form belonging to the Secretariat, which contained name, phone number, date of birth, type of disease and location of residence. After analyzing their charts and considering exclusion criteria, 43 patients were pre-selected to participate in the program. The subjects were contacted by telephone and informed about the program and its goals. Twenty-three individuals showed interest and were selected to participate in the intervention program with aerobic or strength exercises during a 20 week period.

As criteria for inclusion, patients with DM 2, hypertensive or not, of both genders, older than 18, without routine or organized workouts were considered. As exclusion criteria, were considered patients with symptomatic ischemic heart disease or with electrocardiographic signs of ischemia at rest or at TT, pressure instability during TT, kidney diseases, peripheral distal symmetric polyneuropathy, autonomic neuropathy, severe retinopathy, patients with severe osteoarticular diseases, with chronic diseases, drug addicts and people with psychological disorders which prevented the insertion in the proposed study.

As such, by the end of the first phase, the study sample was made up of only 14 volunteers: 4 type 2 diabetics and 10 hypertensive type 2 diabetics (5 men and 9 women). The second phase ended with 11 patients, 4 type 2 diabetics and 7 hypertensive type 2 diabetics (5 men and 6 women).

The minimum size of the sample was defined using the variation coefficient previously obtained for hemoglobin A1c (HbA1c) (16 %) in the literature [15–17] taking into consideration a 15 % variation around the average, with a minimum number of five individuals in each group to verify statistical differences with a 5 % level of significance [18].

All participants selected for the study were met by the Hiperdia Center of Viçosa-MG, and were informed about the methodology, the objectives of the study and signed an informed consent form. Additional file 1: Figure S1 is showing the study flow chart.

### Assessments

All patients were submitted to clinical and cardiological testing with TT in a ramp protocol prior to participation in the study, conducted by a cardiologist in the Center itself. In addition, biochemical tests were conducted before the start of the exercise program, after 10 weeks and at the end of the 20 weeks of training, in accredited laboratories by the Unified Health System (SUS). The tests comprised a glycemic profile (fasting glucose, postprandial glucose, hemoglobin A1c and their blood glucose average), insulin serum, lipid profile (total cholesterol, HDL, LDL and VLDL), and triglycerides. Normality criteria of the assessed blood parameters followed the proposal given by the Brazilian Society of Diabetes [6]. For insulin resistance, the HOMA-IR index proposed by Matthews [19] was calculated. The patients also underwent anthropometric evaluations at the beginning of the program, after 10 weeks and at the end of 20 weeks of training.

Body weight was measured using a Mercy^®^ scale (model LC 200, Brazil, 2010), with the scale ranging from 1 to 200 kilograms and with a 50-g precision. The stature was obtained using a Welmy^®^ stadiometer (model R110, Brazil, 2009) with a scale ranging from 0.8 to 2.00 m with 1 mm accuracy. The body mass index (BMI) was calculated using the formula BMI = body weight (kg)/height (m)^2^. The adopted cutoffs were those recommended by the World Health Organization [20].

The waist, abdomen and hips circumferences were measured using a retractable measuring tape and flexible Proximus^®^ (Rio de Janeiro, Brazil, 2013), with a range of 0–200 cm and 1 mm accuracy. Waist/hip ratio (WHR) were obtained by dividing the perimeter of the waist by the hip and following the classification criteria proposed by the World Health Organization [21].

For all evaluations the methodological recommendations proposed by the International Standards for Anthropometric Assessment (ISAK) [22] were followed, being carried out by two physical education teachers trained using the cited technical manual.

### Exercise programs

The participants were randomly divided into two groups, with distinct exercise programs. One group performed AT and the other ST. In both cases the program lasted 20 weeks and was divided into two 10 week phases, with a frequency of 3 weekly sessions.

Initially, with the purpose of properly adapting the physiological and motor capabilities of the patients, the duration of the main part of the session was initially 20 min, evolving to 30 min in week two and to 40 min in the subsequent week, for both intervention groups.

For both groups, the warm up was performed on equipment like a treadmill, stationary bike, elliptical, or arm ergometer, for 10 min at a max intensity of 50 % of the maximum heart rate (MHR) estimated by the equation MHR = 208 − (0.7 × age) [23]. At the end the cool down was composed of active and passive stretching exercises. The average time of each full session of exercise was between 50 and 60 min beginning from week three.

Exercise procedures were based off of the general guidelines of exercise prescribed for diabetics proposed by the American Diabetes Association [8], the Brazilian Society of Diabetes [6] and for hypertensive individuals proposed by the American College of Sports Medicine [24].

Keeping in mind the safety of the volunteers, capillary blood glucose measurements and BP were taken before, during and after exercise, with daily records of the three measurements for each participant, in addition to the effort perception index (EPI) using the Borg [25] scale during the exercise, for both groups. For blood glucose, measurements were taken using the AccuChek Roche glucometer^®^ Performa (Mannheim, Germany, 2009) while for BP the stethoscope and aneroid sphygmomanometer Premium^®^ (Wenzhou Instrument Co., China, 2014) were used.

All training sessions were individually supervised by physical education professionals. A presence adherence ≥80 % during the training sessions was considered a criterion so that the patient could be included in the final sample.

The main part of the exercise sessions of the two groups had different methodologies. One had the unique execution strength exercises, while the second had aerobic exercises. The ST group followed a sequence of ten exercises: rowing, squat, bench press, rowing with dumbbells, knee extensions with ankle weights, dumbbell shoulder press, dumbbell curls, dumbbell leg extensions, standing calf raises, triceps pull down and abdominal crunches in the first phase of the program. A circuit method was used, but at intervals of 10–15 s between exercises. Seeking an adequate adaptation of neural, joint and muscle systems, the first 2 weeks the volunteers performed 2 sets of 15 repetitions for each exercise and from the third week began to perform 3 sets. The execution of the repetitions of the strength exercises was performed in a continuous, controlled manner with moderate speed and similar durations between concentric and eccentric phases.

Due to the low physical fitness and motor skills of patients, the initial loads of each exercise were set according to the perception of effort, using the scale of 6–20 proposed by Borg [25], and as improvements occurred in the movement pattern and physical conditioning, the weights were adjusted. The scale values used were from 11 to 13, representing moderate effort [26]. We opted for the limitation and control of the training load on strength exercises based on the subjective perception of effort due to the low levels of physical fitness, motor skills and the fact of dealing with high risk patients. Load testing or maximum repetitions would entail an effort inconsistent with participants’ health conditions, which would raise the risk of adverse events. Therefore, the training loads were adjusted according to the improvement in the physical fitness and motor skills of the participants, and even with load adjustments the perception of the effort of the participants always remained between 11 and 13.

During the second phase of the program there was a change in the exercise program. Participants kept the sequence of 10 exercises in a circuit with a frequency of 3 weekly sessions and keeping the muscle groups exercised during the previous routine. Repetitions were changed to 12 per exercise and participants followed the following sequence: front lat pull down, unilateral squats, barbell bench press, unilateral leg raise (standing), lateral/front raises, standing calf raises, standing biceps cable curl, cable triceps pushdown, and unilateral cross crunch. During the first week of the second phase of the program, the participants performed 2 series, moving to 3 series on the second week until the end of the program.

For the AT group, with an aim of proper physiological and motor adaptation, the initial duration of the main part of the session was 20 min in the first week, 30 min in the second week, and 40 min the subsequent weeks. The training was performed on the treadmill, exercise bikes, elliptical and an upper body cycle ergometer. Initially there was a proposal to control training intensity through the MHR percentage, setting it at 60 % for the main part of the training. However, due to some patient’s use of adrenergic beta-blockers to control BP, the decision was made to use the subjective effort perception scale, proposed by Borg [25], which was used for the control of training loads of these patients. For diabetic patients who did not show symptoms of SH or use beta blockers, the initial plan was utilized.

The lower and upper limit range from 6 to 20, respectively, was adopted for the control of the training session intensity. Another justification for the use of this methodology of intensity control was low physical fitness, motor skills and participant mobility. The scale values used were from 11 to 13, representing moderate effort [26], and were in accordance with the indications for prescribing exercises proposed by the Brazilian Society of Diabetes [6] and American Diabetes Association [8].

In the second phase of the program a change was made to the intensity of training, from 60 to 70 % of the MHR formula stipulated by Tanaka et al. [23]. For patients who use beta-blockers, the scale of perceived exertion [25] was used. During the first week of the second phase of the program the patients performed 30 min in the main part of the session, achieving 40 min during the second week until the end of the program. Although it is still considered a moderate intensity on the Borg scale [25], the change from 60 to 70 % MHR can be considered a real change of intensity because the participants exercised at a higher percentage of MHR, but kept EPI between 11 and 13. Even patients on beta blockers reached this level of MHR.

### Statistical analysis

For the presentation of data, descriptive statistics was used. For the analysis of the distribution of data the Shapiro–Wilk test was used. For the analysis of the responses of all analyzed variables during the three periods (baseline, 10 weeks and 20 weeks), as well as verifying changes over time and the differences between the groups, a mixed ANOVA model was used. The effect size was calculated for the magnitude of observed effect [27]. A significance level of p < 0.05 was considered.

The standard deviation (SD) and intraclass correlation coefficient (*r*) of the initial assessment (baseline) and of the 20 weeks were used to estimate the standard error of measurement (SEM). The formula *SEM* = *SD* × *√1* − *r* was used. The Minimum Detectable Change (MDC) was used to define the smallest change that probably reflects a real change rather than a measurement error. The MDC is more often based on a 90 % confidence interval (*z* = 1.65) [28].

The MDC_90_ was calculated using the formula MDC_90_ = 1.65 × SD × √(2 × [1 − *r*]), where 1.65 is the value of z of two tails for a 90 % confidence interval, SD is the standard deviation, √2 represents the variation of the two measurements, and *r* is the intraclass correlation coefficient. All analyses were performed using the software SPSS (SPSS Inc^®^, version 20).

## Results

Tables 1 (ST) and 2 (AT) present the results of the comparison means of anthropometric data of the two groups at three periods during the intervention: baseline, 10 and 20 weeks.

**Table 1:** Comparisons of the anthropometric variables at baseline, 10 and 20 weeks for the ST group (n = 5)

	Baseline	10 weeks	20 weeks
Age (years)	57 ± 12	**–**	**–**
Body weight (kg)	71 ± 5	71 ± 6	71 ± 5
Height (m)	1.62 ± 0.09	**–**	**–**
BMI (kg/m^2^)	27 ± 3	27 ± 4	27 ± 4
Waist Circ. (cm)	93 ± 4	97 ± 9	95 ± 7
Abdomen Circ. (cm)	98 ± 5	99 ± 7	99 ± 6
Hip Circ. (cm)	97 ± 2	98 ± 7	98 ± 5
WHR	0.95 ± 0.05	0.98 ± 0.06	0.97 ± 0.08

Results presented in mean and standard deviation values

Significance level p < 0.05

*BMI* body mass index, *WHR* waist hip ratio, *Circ.* circumference

**Table 2:** Comparisons of the anthropometric variables at baseline, 10 and 20 weeks for the AT group (n = 6)

	Baseline	10 weeks	20 weeks
Age (years)	54 ± 9	**–**	**–**
Body weight (kg)	94 ± 31	93 ± 30	91 ± 29
Height (m)	1.60 ± 0.1	**–**	**–**
BMI (kg/m^2^)	36 ± 10	35 ± 9	35 ± 9
Waist Circ. (cm)	110 ± 24	108 ± 23	107 ± 20
Abdomen Circ. (cm)	116 ± 25	114 ± 22	113 ± 21
Hip Circ. (cm)	111 ± 13	113 ± 10	108 ± 9
WHR	0.98 ± 0.11	0.95 ± 0.14	99 ± 0.16

Results presented in mean and standard deviation values

Significance level p < 0.05

*BMI* body mass index, *WHR* waist–hip ratio, *Circ*. circumference

The anthropometrical variable behavior analysis did not show statistically significant changes between the periods for either groups. In addition, the analysis undertaken showed no interaction between the times and type of exercise or differences between the types of training on the behavior of the variables.

For hip circumference, the analyses showed no significant changes between periods in either groups (p = 0.464), but the hip circumference averages differed in the three periods evaluated (p = 0.02) between the groups. The hip circumference averages observed were significantly higher in the AT group when compared with the ST group at baseline (p = 0.04), 10 weeks (p = 0.02) and 20 weeks (p = 0.05).

The blood variable comparisons results are shown in Tables 3 (ST) and 4 (AT). For hemoglobin A1c (HbA1c) significant changes were observed over the studied periods (p = 0.001), represented by a greater effect size *r* = 0.76, but there were no significant differences in the average HbA1c values between the two groups (p = 0.743). No significant interactions were found between the periods and type of exercise (p = 0.489), that is, changes in the average HbA1c were equivalent in the two types of exercises. The reductions in the averages of HbA1c occurred between the baseline and 10 weeks (p = 0.03), and between the baseline and 20 weeks (p = 0.01), with ST group starting with the highest average value and finalizing the exercise program with average value lower than the AT group.

**Table 3:** Comparisons of the biochemical variables at baseline, 10 and 20 weeks for the ST group (n = 5)

	Baseline	10 weeks	20 weeks
Fasting glycemic levels (mg/dL)	174 ± 92	129 ± 46	157 ± 86
Postprandial glycemic levels (mg/dL)	187 ± 76	142 ± 96	187 ± 100
HbA1c (%)	9.2 ± 1.9^a,b^	7.9 ± 1.2^a^	7.4 ± 0.9^b^
Average glycemic level (mg/dL)	217 ± 56^c,d^	179 ± 36^c^	165 ± 29^d^
Total cholesterol (mg/dL)	174 ± 49	155 ± 23	169 ± 40
LDL (mg/dL)	106 ± 51	83 ± 8	93 ± 25
VLDL (mg/dL)	25 ± 20	27 ± 14	28 ± 17
HDL (mg/dL)	45 ± 3	45 ± 3	48 ± 8
Triglycerides (mg/dL)	128 ± 97	136 ± 72	127 ± 55
Seric Insulin (μUi/mL)	30 ± 24	23 ± 21	18 ± 14
HOMA-IR	13 ± 14	6 ± 4	5 ± 4

Results presented in mean and standard deviation values

Significance level p < 0.05

*HbA1c* hemoglobin A1c

^a,c^p = 0.03 (*baseline and 10* *weeks*)

^b,d^p = 0.01 (*baseline and 20* *weeks*)

**Table 4:** Comparisons of the biochemical variables at baseline, 10 and 20 weeks for the AT group (n = 6)

	Baseline	10 weeks	20 weeks
Fasting glycemic levels (mg/dL)	187 ± 107	153 ± 56	146 ± 66
Postprandial glycemic levels (mg/dL)	225 ± 172	161 ± 43	158 ± 82
HbA1c (%)	8.6 ± 2.5^a,b^	7.2 ± 1.7^a^	7.5 ± 1.7^b^
Average glycemic levels (mg/dL)	202 ± 74^c,d^	161 ± 51^c^	171 ± 49^d^
Total cholesterol (mg/dL)	193 ± 26	187 ± 23	167 ± 12
LDL (mg/dL)	103 ± 29	99 ± 23	74 ± 27
VLDL (mg/dL)	41 ± 22	41 ± 12	49 ± 13
HDL (mg/dL)	53 ± 11	49 ± 14	49 ± 15
Triglycerides (mg/dL)	183 ± 113	219 ± 112	177 ± 80
Seric Insulin (μUi/mL)	14 ± 9	13 ± 6	11 ± 9
HOMA-IR	5 ± 4	5 ± 2	4 ± 3

Results presented in mean and standard deviation values

Significance level p < 0.05

*HbA1c* hemoglobin A1c

^a,c^p = 0.03 (*baseline and 10* *weeks*)

^b,d^p = 0.01 (*baseline and 20* *weeks*)

The average blood glucose values changed significantly over the studied periods (p = 0.001), with *r* = 0.76 representing a greater magnitude of effect. However, there were no significant differences in mean values of this parameter between the two groups (p = 0.742). Also, no significant interactions were observed between the periods and the type of exercise (p = 0.487), even though the changes occurred similarly in both groups. The reductions in blood glucose average values occurred between the baseline and 10 weeks (p = 0.03) and between the baseline and 20 weeks (p = 0.01), with the ST group starting with the highest average value and finalizing the exercise program with an average value lower than the AT group. All other variables evaluated showed no statistically significant changes during the exercise program.

The results of the MDC_90_ for HbA1c is presented in Table 5. For the average HbA1c, which showed statistically significant difference in the averages of the two groups, 40 % of the subjects belonging to the ST group and 33 % belonging to the AT group reached minimal changes in their averages.

**Table 5:** Subjects who reached the minimum detectable change criterion in glycemic control at 90 % confidence (MDC_90_)

	MDC_90_	n (%)
HbA1c (%)		
Strength	2.52	40
Aerobic	1.73	33
Average glycemic level (mg/dL)		
Strength	72.23	40
Aerobic	49.77	33

*SD* standard deviation, *MDC*_*90*_ minimum detectable change at 90 % confidence, *n (%)* percentage of each group who attained minimum detectable change values

Additional file 2: Figure S2 presents the average HbA1c individual behavior throughout the 20 week intervention for the ST and AT groups. It was found that individuals who obtained greater reductions in the average HbA1c were the ones who presented greater averages at the beginning of the program.

## Discussion

The objective of the present study was to evaluate and compare the effects of aerobic exercise and strength training programs on glycemic control in patients with DM 2 treated through the public health system. Regular physical exercise is an important component in the control and treatment of DM 2 [9], promoting benefits on insulin sensitivity, plasma glucose homeostasis, increase in daily energy expenditure [10], cardiovascular risk reduction, and contribution to weight loss and general well-being [8].

Even without statistically significant changes in any anthropometric variables (Tables 1, 4), Tables 2 and 5 present statistically significant differences in average HbA1c and the estimated average glucose in both the ST and AT groups for patients who completed 20 weeks of training. Although the reductions were statistically non-significant, the average fasting glucose and postprandial glucose levels presented relevant reductions, except for the postprandial glucose of the ST group.

Even considering that the fasting glycemic levels value is insufficient for monitoring glycemic control in subjects with DM, this measure has important clinical relevance, because it reflects a specific time point of these patients’ glycemic levels [6]. In recent years the postprandial hyperglycemia has been recognized as an independent risk factor for the development of cardiovascular complications and oxidative stress in patients with DM [6], and structured exercise programs have shown to play an important role in glycemic control during the day, causing increased sensitivity to insulin for up to 48 h after the workout, which can assist in preventing hyperglycemic spikes [29].

In absolute numbers, the average reductions in HbA1c were 1.3 and 1.8 % for the ST group and 1.4 and 1.1 % for the AT group in comparison to baseline values with 10 and 20 weeks, respectively. The estimated average glucose values reduced significantly in comparison to the baseline with 10 and 20 weeks. For the ST group, the reductions were 18 and 24 %, while for the AT group these reductions were 20 and 15 %.

Studies have shown that structured aerobic exercise or resistance exercises reduce HbA1c levels, on average, about 0.6 % in patients with DM 2 [8]. The reduction in HbA1C levels is associated with a decreased risk of cardiovascular events and microvascular complications, so regular physical exercise over time can assist in effective glycemic control, reducing the risk of vascular complications [13]. In absolute numbers, a 1 % reduction in HbA1c value is associated with a reduction between 15 and 20 % of the risk of cardiovascular problems and 37 % of the risk of microvascular complications [15]. Values higher than the percentage above quoted were found by the present study.

Interventions using structured exercise programs with a minimum of 8 weeks showed positive results in glycemic control in DM patients, commonly without significant changes in BMI [8, 11, 12], which corroborates the results of the present study. Even with statistically or clinically significant reductions in glycemic control parameters, anthropometric parameters such as body weight, BMI, WHR and circumferences showed no relevant changes over the 20 weeks of training, as shown in Tables 1 and 4. This result can be explained by not having caloric restrictions planned and, perhaps, the volume and intensity of the exercises prescribed. According to Sigal et al. [12], most of the studies which succeeded in the loss and control of body weight involved the combination of exercise with diet and behavioral changes, and interventions with only exercise tended to produce modest changes in body weight, and the volume of exercise for weight loss was greater than what is needed to promote glycemic control and cardiovascular health.

The blood analysis parameters related to long-term glucose control (HbA1c and estimated average glycemic levels) demonstrates a similarity in the responses submitted by individuals of the two intervention groups. As occurred in the comparisons of the general averages over the 20 weeks of intervention, the analysis by MDC_90_ to HbA1c, even though there were different values between the groups, did not show differences between them when the number of subjects who attained a real reduction in these values were considered.

Individually, those who reached values above the MDC_90_ were those who presented a higher value on the basis of the variable analyzed which is justified, according to Haley and Fragala-Pinkhan [30], subjects that begin with higher values (worst metabolic control) in a given evaluation tend to have greater ability to achieve real changes than those that present smaller values (better metabolic control).

The use of the MDC_90_ allows the insertion of a new type of behavior analysis for the glycemic metabolism with the intervention through physical exercise, so that answers can be considered important from the point of view of clinical control, individually, regardless of the level of significance of the comparisons.

The present study presented the results of exercise programs as adjuvant therapy to control the DM 2 in secondary healthcare. Although clinically relevant results were found in the improvement of the metabolic control of participants of both groups, the study presented limitations with the reduced sample size due to losses from adherence and exclusion criteria. It is important to consider that the Hiperdia is a center of secondary health attention, linked to the Brazilian Public Health System. The Hiperdia participants are people from low socioeconomic status, low human development index, low educational background and very low familiar income. These participants have intellectual difficulties in understanding, and as consequence to adherence, some therapies, as physical activity and nutritional orientations, as an important part of DM treatment. Some of the patients cannot participate in the exercise interventions because of conflicting working hours, while others, living far from the center have not enough money for the public transport 3 days a week for 20 weeks to get to the training sessions, which contributes directly to the small sample size.

Another limiting factor would be the most pronounced differences in body weight and BMI between the two groups at baseline. However, the randomization process was done, but the reduction of participant number by loss of adherence or clinical complications by many patients, inevitably, increasing these differences. Initially both groups composed by 23 selected patients for the intervention sessions showed smaller differences in body mass and BMI than the groups completing 20 weeks of intervention sessions. However, despite these showed average differences between groups at the end of the study intervention, it was not enough to produce statistically significant differences.

It is important to stress that the option to use estimated maximal heart rate percentage to training control it is a procedure recommended by guidelines as Brazilian Diabetes Society [6] and American College Sports Medicine joint with American Diabetes Association [31]. It is a tool habitually used to control interventions in the therapeutic practice with patients with a range of health problems, among them diabetes and hypertension. We are aware that the best method to evaluate oxygen uptake, anaerobic threshold and maximal heart rate is through progressive maximal exercise testing with a gas analyzer. However, analyzing the Hiperdia participants characteristics (low physical fitness, limited motor behavior, low ergometer habituation, medicine use, and all included risk factor), and discussing with center cardiologists, we did the option to not adopt this type of test. A progressive maximal exercise testing with patients with all cited conditions probably will produce false positive results and will not introduce additional information to the performed test, considering the study objective, but could introduce complications and increase patients stress and burden. Moreover, the test applied before patients starting the exercise program was exclusively to evaluate physical activity readiness, in the clinical perspective.

It is important to consider these results as a start point of a free and new supervised exercise program methodology in the Brazilian Public Health System, specifically in the secondary health attention with high risk patients. We strongly believe that the implantation of this program in other centers will create new opportunities for a large number of patients increasing the recruitment and participation adherence.

## Conclusion

The data submitted demonstrated that using both aerobic exercise and ST can aid in the metabolic control of patients with DM 2, even without changes in anthropometry or caloric intake control. The characteristics of patients seen by the Hiperdia Center of Viçosa and that composed the study sample, age and the high prevalence of physical inactivity may have acted as an ineffective metabolic control and may have contributed to more pronounced reductions than the results of some previous studies.

Both the aerobic exercises and weight training, prescribed from the proposed methodology, can effectively help the metabolic control of patients seen in secondary healthcare and that these individuals should be encouraged to enter into supervised exercise programs involving both types of exercises, with a suggestion to increase the frequency to 5 days a week, in addition to a stricter dietary control. To our acknowledgement this is the first study to use MDC as outcome measure for exercise intervention as adjunct therapy to glycemic control for diabetic patients, our results can be useful as reference for future studies or intervention, but with caution given the small sample size. New studies are warranted to verify the reproducibility of our findings.

## Additional files

10.1186/s13098-016-0123-2 Study flow chart. AT = aerobic training; ST = strength training.
10.1186/s13098-016-0123-2 Behavior of individual HbA1c averages over the 20 week training. The dotted line represents the acceptable value by the Brazilian Society of Diabetes [6] for HbA1c (7 %); ST = strength training; AT = aerobic training.

## Abbreviations

AT: aerobic training
BMI: body mass index
BP: blood pressure
DM: diabetes *mellitus*
DM 2: type 2 diabetes *mellitus*
EPI: effort perception index
HbA1c: hemoglobin A1c
HDL: high-density lipoprotein cholesterol
LDL: low-density lipoprotein cholesterol
MDC: minimum detectable change
MDC_90_: minimum detectable change on a 90 % confidence interval
MG: Minas Gerais state
MHR: maximum heart rate
SH: systemic hypertension
ST: strenght training
SUS: unified health system
TT: treadmill test
VLDL: very low-density lipoprotein cholesterol
WHR: waist–hip ratio

## References

[1] Cheng YJ, Imperatore G, Geiss LS (2013). Secular changes in the age-specific prevalence of diabetes among US adults: 1988–2010. Diabetes Care.

[2] International Diabetes Federation. IDF Diabetes Atlas. 2013. Sixty Edition.

[3] Lorber D (2014). Importance of cardiovascular disease risk management in patients with type 2 diabetes *mellitus*. Diabetes Metab Syndr Obes.

[4] Almeida-Pititto B, Dias ML, Moraes ACF, Ferreira SRG, Franco DR, Eliaschewitz FG (2015). Type 2 diabetes in Brazil: epidemiology and management. Diabetes Metab Syndr Obes.

[5] Chittleborough CR, Grant JF, Phillips PJ, Taylor AW (2007). The increasing prevalence of diabetes in South Australia: the relationship with population ageing and obesity. Public Health.

[6] Sociedade Brasileira de Diabetes. Diretrizes da Sociedade Brasileira de Diabetes 2013–2014. 2014.

[7] Matthews L, Kirk A, Mutrie N (2014). Insight from health professionals on physical activity promotion within routine diabetes care. Pract Diabetes.

[8] Standards of Medical Care in Diabetes-2015. American Diabetes Association. Diabetes Care. 2015;38(Suppl 1):1–94.

[9] Moura BP, O’Neill HM, Amorim PR (2015). Can an aerobic exercise program influence sedentary behavior and moderate-vigorous physical activity in patients with type 2 diabetes?. Ann Sports Med Res.

[10] Moura BP, Natali AJ, Marins JCB, Amorim PRS (2011). Different approaches of physical training used in the management of type 2 diabetes: a brief systematic review of randomised clinical trials. BrJ Diabetes Vasc Dis.

[11] Boulé NG, Haddad E, Kenny GP, Wells GA, Sigal RJ (2001). Effects of exercise on glycemic control and body mass in type 2 diabetes *mellitus*. JAMA.

[12] Sigal RJ, Kenny GP, Wasserman DH, Castaneda-Sceppa C (2006). White RD Physical activity/exercise and type 2 diabetes: a consensus statement from the American Diabetes Association. Diabetes Care.

[13] Rydén L, Grant PJ, Anker SD, Berne C, Cosentino F, Danchin N (2013). ESC guidelines on diabetes, pre-diabetes, and cardiovascular diseases developed in collaboration with the EASD: the Task Force on diabetes, pre-diabetes, and cardiovascular diseases of the European Society of Cardiology (ESC) and developed in collaboration with the European Association for the Study of Diabetes (EASD). Eur Heart J.

[14] Sluik D, Buijsse B, Muckelbauer R, Kaaks R, Teucher B, Johnsen NF (2012). Physical activity and mortality in individuals with diabetes mellitus. A prospective study and meta-analysis. JAMA.

[15] Sigal RJ, Kenny GP, Boulé NG, Wells GA, Prud’homme D, Fortier M (2007). Effects of aerobic training, resistance training, or both on glycemic control in type 2 diabetes. Ann Intern Med.

[16] Church TS, Blair SN, Cocreham S, Johannsen N, Johnson W, Kramer K (2010). Effects of aerobic and resistance exercise training on hemoglobin A1c levels in patients with type 2 diabetes: a randomized controlled trial. JAMA.

[17] Moura BP, Amorim PRS, Silva BPP, Franceschini SCC, Reis JS, Marins JCB (2014). Effect of a short-therm exercise program on glycemic control measured by fructosamine test in type 2 diabetes patients. Diabetol Metab Syndr.

[18] Hulley SB, Cummings SR. Estimating sample size and power. In: Designing clinical research. Baltimore, MD: Williams and Wilkins; 1988:148(Appendlix 13A):215.

[19] Matthews DR, Hosker JP, Rudenski AS, Naylor BA, Treacher DF, Turner RC (1985). Homeostasis model assessment: insulin resistance and beta-cell function from fasting plasma glucose and insulin concentrations in man. Diabetologia.

[20] World Health Organization (WHO) (2003). Diet, nutrition and the prevention of chronic diseases. World Health Organ Tech Rep Ser.

[21] World Health Organization (WHO) (2008). Waist circumference and waist–hip ratio: report of a WHO expert consultation.

[22] Marfell-Jones M, Olds T, Stewart A, Carter L (2006). International standards for anthropometric assessment.

[23] Tanaka H, Monahan KD, Seals DR (2001). Age-predicted maximal heart revisited. J Am Coll Cardiol.

[24] Pescatello LS, Franklin BA, Fagard R, Farquhar WB, Kelley GA, Ray CA (2004). Exercise and hypertension. American College of Sports Medicine position stand. Med Sci Sports Exerc.

[25] Borg G (1982). Psychophysical bases of perceived exertion. Med Sci Sports Exerc.

[26] American College of Sports Medicine. Perceived Exertion. 2001. https://www.acsm.org/docs/current-comments/perceivedexertion. Accessed 24 Nov 2014.

[27] Field A (2009). Discovering statistics using SPSS: and sex and drugs and rock’n’roll.

[28] Steffen T, Seney M (2008). Test–retest reliability and minimal detectable change on balance and ambulation tests, the 36-item short-form health survey, and the unified Parkinson disease rating scale in people with parkinsonism. Phys Ther.

[29] van Dijk JW, Manders RJF, Tummers K, Bonomi AG, Stehouwer CDA, Hartgens F (2012). Both resistance- and endurance-type exercise reduce the prevalence of hyperglycaemia in individuals with impaired glucose tolerance and in insulin-treated and non-insulin-treated type 2 diabetic patients. Diabetologia.

[30] Haley SM, Fragala-Pinkham MA (2006). Interpreting change scores of tests and measures used in physical therapy. Phys Ther.

[31] Colberg SR, Albright AL, Bissmer BJ, Braun B, Chasan-Taber L, Fernhall B (2010). Exercise and type 2 diabetes: American College of Sports Medicine and the American Diabetes Association: joint position statement: exercise and type 2 diabetes. Med Sci Sports Exerc.

