# Supplementary figures and images for: Effects of different exercise programs and minimal detectable changes in hemoglobin A1c in patients with type 2 diabetes
Source: Diabetol Metab Syndr. 2016 Feb 16;8:13. doi: 10.1186/s13098-016-0123-y (PMC4756416; doi:10.1186/s13098-016-0123-y)

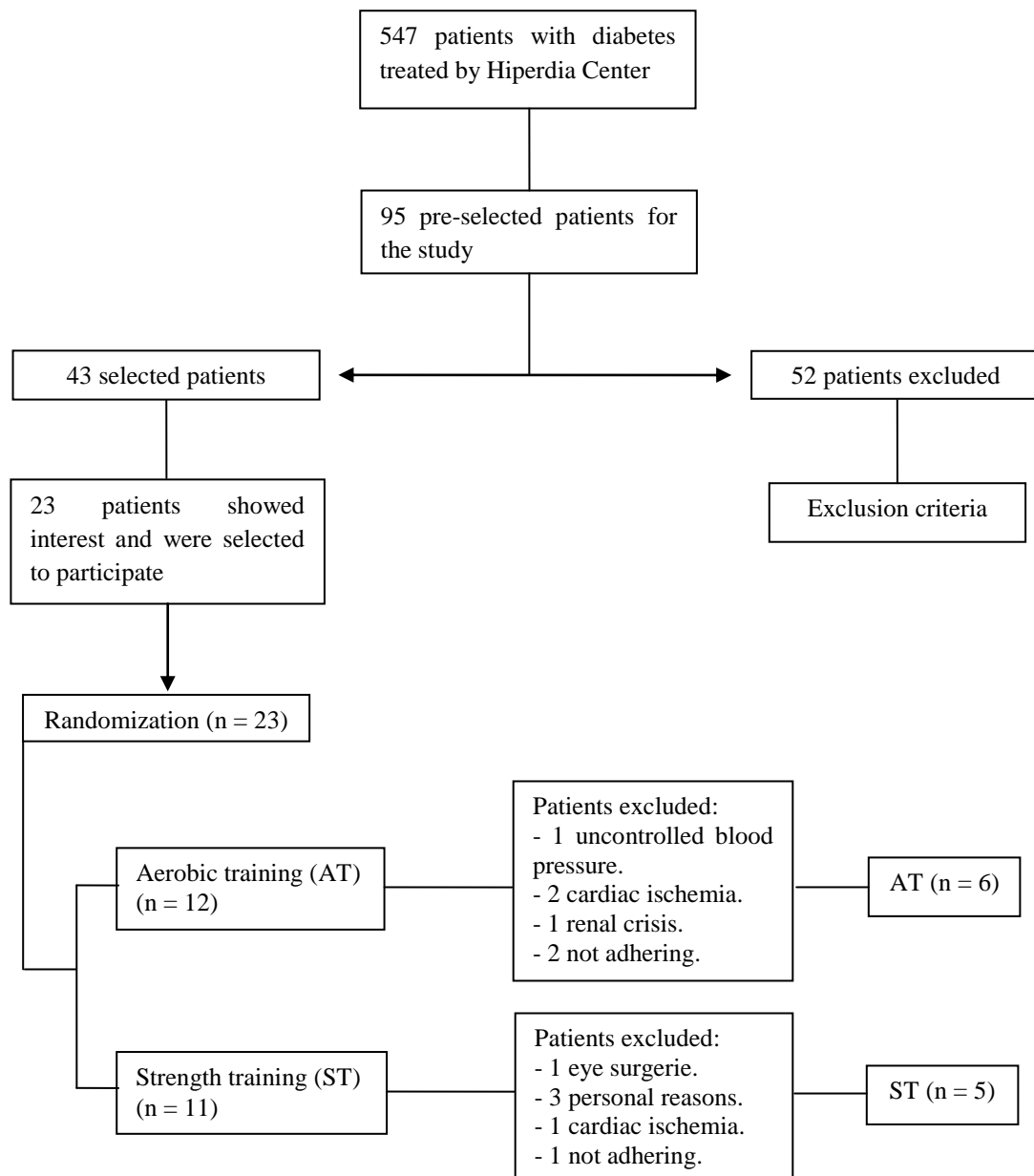

Figure 1. Study flow chart. AT = aerobic training; ST = strength training.

Supplement: Supplementary file 1 — 10.1186/s13098-016-0123-2 Study flow chart. AT = aerobic training; ST = strength training. [file 13098_2016_123_MOESM1_ESM.pdf]
