# Supplementary material for: Effects of different exercise programs and minimal detectable changes in hemoglobin A1c in patients with type 2 diabetes
Source: Diabetol Metab Syndr. 2016 Feb 16;8:13. doi: 10.1186/s13098-016-0123-y (PMC4756416; doi:10.1186/s13098-016-0123-y)
Supplement: Supplementary file 2 — 10.1186/s13098-016-0123-2 Behavior of individual HbA1c averages over the 20 week training. The dotted line represents the acceptable value by the Brazilian Society of Diabetes [6] for HbA1c (7 %); ST = strength training; AT = aerobic training. [file 13098_2016_123_MOESM2_ESM.pdf]

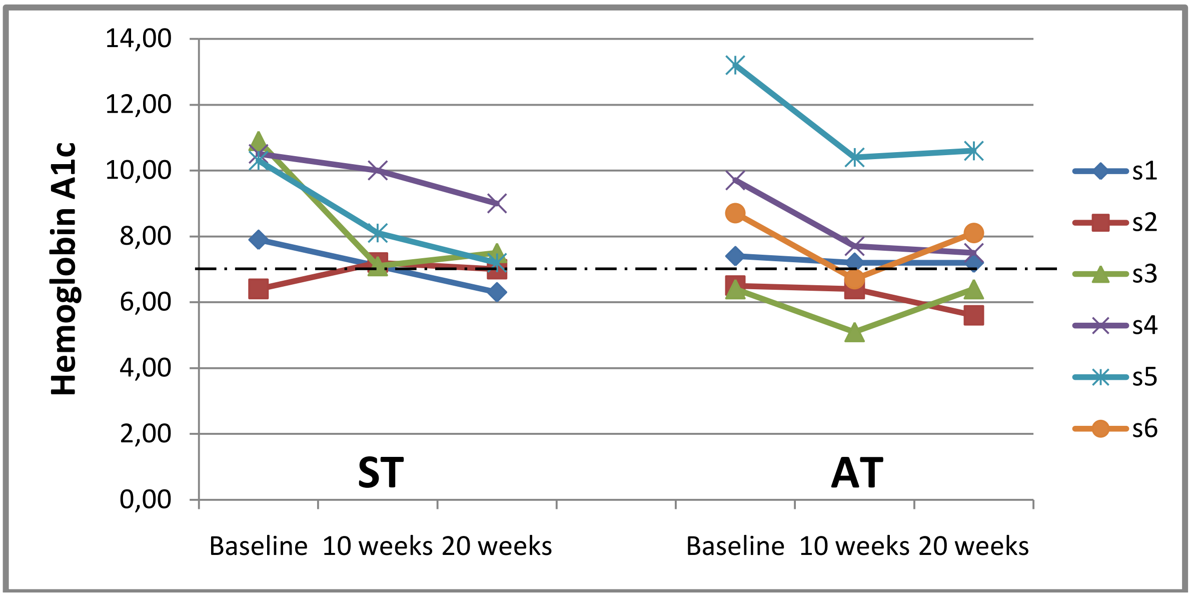

Figure 2. Behavior of individual HbA1c averages over the 20 week training. The dotted line represents the acceptable value by the Brazilian Diabetes Society (SBD, 2014) for HbA1c (7%); ST = strength training; AT = aerobic training.
